# Immunohematologic Biomarkers in COVID-19: Insights into Pathogenesis, Prognosis, and Prevention

**DOI:** 10.20411/pai.v8i1.572

**Published:** 2023-06-26

**Authors:** David R. Sweet, Michael L. Freeman, David A. Zidar

**Affiliations:** 1 Case Western Reserve University School of Medicine, Cleveland, OH; 2 Division of Infectious Diseases and HIV Medicine, Case Western Reserve University, Cleveland, OH; 3 Harrington Heart and Vascular Institute, University Hospitals Cleveland Medical Center, Cleveland, OH; 4 Cardiology Section, Louis Stokes Cleveland Veterans Affairs Medical Center, Cleveland, OH; 5 Center for Global Health and Diseases, Department of Pathology, Case Western Reserve University School of Medicine, Case Western Reserve University, Cleveland, OH

**Keywords:** SARS-CoV-2, COVID-19, biomarkers, multiomics, immunophenotyping, prevention

## Abstract

Coronavirus disease 2019 (COVID-19) has had profound effects on the health of individuals and on healthcare systems worldwide. While healthcare workers on the frontlines have fought to quell multiple waves of infection, the efforts of the larger research community have changed the arch of this pandemic as well. This review will focus on biomarker discovery and other efforts to identify features that predict outcomes, and in so doing, identify possible effector and passenger mechanisms of adverse outcomes. Identifying measurable soluble factors, cell-types, and clinical parameters that predict a patient's disease course will have a legacy for the study of immunologic responses, especially stimuli, which induce an overactive, yet ineffectual immune system. As prognostic biomarkers were identified, some have served to represent pathways of therapeutic interest in clinical trials. The pandemic conditions have created urgency for accelerated target identification and validation. Collectively, these COVID-19 studies of biomarkers, disease outcomes, and therapeutic efficacy have revealed that immunologic systems and responses to stimuli are more heterogeneous than previously assumed. Understanding the genetic and acquired features that mediate divergent immunologic outcomes in response to this global exposure is ongoing and will ultimately improve our preparedness for future pandemics, as well as impact preventive approaches to other immunologic diseases.

## INTRODUCTION

**Figure 1.**
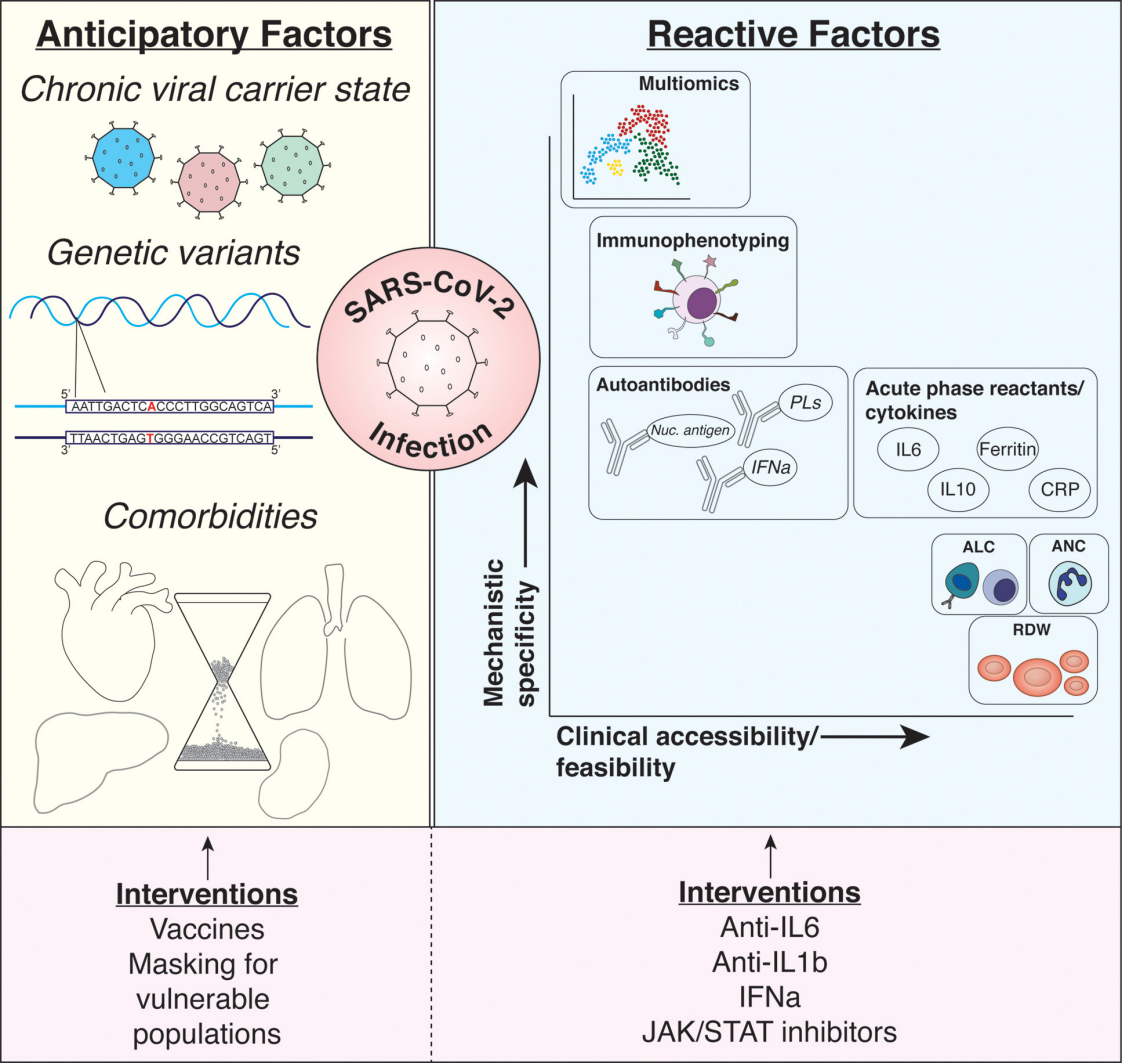
**Summary of discussed biomarkers.** *Anticipatory* (ie, pre-infection) and *reactive* (ie, post-infection) biomarkers of COVID-19 infection risk and outcomes are described. Among the characteristics creating risk for infection and poor outcomes prior to SARS-CoV-2 infection include being a chronic carrier of a multitude of different viruses (eg, Epstein-Barr virus and cytomegalovirus), genetic variants predisposing individuals to impaired immunologic responses, and chronic comorbid conditions (eg, aging, heart disease, metabolic disease). Interventions possible at this stage of *biomarker* include preventative strategies protecting at-risk populations including vaccine and mask utilization. While numerous reactive biomarkers have been identified as discussed in the manuscript, each provides varying levels of mechanistic insight or utility in adapting such biomarkers to large clinical populations (shown with x and y axes). Among the most promising biomarkers discussed include simple complete blood count parameters (eg, ALC, ANC, and RDW), soluble serologic factors such as cytokines and acute phase reactants, deep profiling of immunophenotypes (through flow cytometric analysis of immune surface receptor expression), and multi-omic analyses using proteomics, metabolomics, and immunophenotypes. Targeting these factors has been more aggressive during the pandemic with immunomodulatory agents (eg, anti-IL6, anti-IL1β agents) with varying levels of success. Abbreviations: ALC, absolute lymphocyte count; ANC, absolute neutrophil count; CRP, C-reactive protein; IFNα, interferon alpha; IL1β, interleukin-1 beta; IL6, interleukin-6; IL10, interleukin-10; Nuc. antigen, nuclear antigens; PLs, phospholipids; RDW, red blood cell distribution width; SARS-CoV-2, severe acute respiratory syndrome coronavirus-2.

Early in the first wave of COVID-19, it was observed that a robust immune response to infection could be responsible for some of the devastating consequences of the disease. Predicting patient outcomes based on their acute response to infection, therefore, became of utmost importance in early biomarker studies. From these early studies and those that followed, numerous serum markers were identified with sophistication ranging from routine laboratory parameters to unbiased, computationally demanding multi-omic studies (Figure 1).

### Clinical Laboratory Measurements Illustrating the Inflammatory Milieu

Among the first identified prognostic markers were those pulled from routine, repeated clinical laboratory studies such as complete blood counts (CBC), common acute phase reactants, and targeted cytokine assays. Unsurprisingly, numerous CBC parameters are perturbed in the early response to severe acute respiratory syndrome coronavirus-2 (SARS-CoV-2) infection, many of which were found to be predictors of pulmonary disease severity (eg, hypoxia, intensive care unit (ICU) admission, end-organ damage, mechanical ventilation, mortality). For instance, greater leukocytosis is almost universally associated with poor outcomes. In univariable analyses comparing outcomes within infected individuals, there was an odds ratio (OR) of in-hospital death of 6.6 when white blood cell (WBC) counts were greater than 10 x 10^9^/L and significantly elevated levels of WBC were seen when comparing patients who required ICU care to those who did not, between survivor vs non-survivor groups, and between individuals with acute respiratory distress syndrome (ARDS) and those without [1–3]. The predictive value of admission WBC count on survival holds after adjustment for age, sex, and comorbid cardiometabolic factors and in meta-analyses from the first wave of COVID-19 [4–6]. WBC elevation largely reflects neutrophilia as a surrogate of the acute response to pathogen-associated molecular patterns (PAMPs) and damage-associated molecular patterns (DAMPs) elaborated during COVID-19. Neutrophilia is seen on presentation of many patients infected with SARS-CoV-2, with elevated and sustained levels predicting worse outcomes [1, 3, 7, 8].

Lymphopenia is also a powerful predictor of severity, development of ARDS, need for ICU care, and death in infected individuals [1, 3, 9, 10]. In a meta-analysis encompassing 6449 COVID-19+ patients, low absolute lymphocyte count (ALC) was associated with an OR of 3.33 for having poor outcomes [2, 11]. Interestingly, lymphopenia at admission that quickly reaches its nadir and begins to resolve early in the disease course is associated with more favorable outcomes than individuals whose ALC fails to recover [2, 12–15]. Whether this lymphopenic response is a physiologic reporter of disease severity only or whether it contributes to immune failure remains an open question.

Multiple mechanisms whereby viral infection (SARS-CoV-2 and otherwise) leads to peripheral lymphocyte depletion have been proposed. These include via cell death (eg, apoptosis, pyroptosis), induction of anergy, inhibited lymphopoiesis via cytokine release, and/or exaggerated sequestration in secondary lymphoid tissue or end organs [16–18]. The associated stress response via catecholamines, cortisol, and other neurohormonal effects may also contribute to lymphopenia. Asymptomatic individuals with SARS-CoV-2-infection appear to maintain lymphocyte levels [19]. Ultimately, however, the lack of large datasets recording pre-infection lymphocyte counts has hampered full dissection of these interactions. There is likely an interplay between these 2 mechanisms that results in a cycle of lymphocyte destruction and dysfunction. The implications of this are discussed further below.

Since neutrophilia and lymphopenia are associated with poor outcomes in COVID-19 and other infections, the neutrophil-to-lymphocyte ratio (NLR) has been used to gauge the magnitude of an immune response [20–22]. Multivariate retrospective analysis of COVID-19+ patients over the age of 60 years demonstrated a significant increase in hazard ratio for NLR on course severity and a prospective analysis revealed a strong predictive power for NLR in predicting general clinical deterioration, shock, and death [10, 23].

COVID-19 has been associated with alterations in other hemogram parameters including thrombocytopenia, anemia, and red blood cell distribution width (RDW) [2, 7, 9–11, 24–28]. For instance, an elevated RDW predicts poor outcomes, including mortality, and often reflects an antecedent imbalance between pro-inflammatory and regulatory plasma cytokine levels [29]. Change in red cell size could occur through multiple mechanisms including decreasing RBC lifespan, immune-mediated destruction, impaired erythropoiesis secondary to cytokine-mediated inhibition of progenitors, and changes in iron metabolism [30–32].

In addition to cellular parameters of the acute inflammatory response, commonly tested soluble factors have been identified as valuable biomarkers of COVID-19 status and progression. Among these, acute phase reactants such as C-reactive protein (CRP), serum amyloid A (SAA), procalcitonin (PCT), and ferritin have been shown to consistently associate with severe clinical presentation and mortality [1–3, 9, 11, 15, 28, 33–37]. Similarly, so-called negative acute phase reactants such as transferrin and albumin have been noted to be inversely associated with poor outcomes and can predict clinical course [38, 39]. The temporal response of albumin also appears to predict negative outcomes: individuals with hypoalbuminemia with a larger change in albumin on a second draw had fewer complications compared to those with hypoalbuminemia and a small change in albumin on repeat check [40]. Further, combining this finding with the presence of lymphopenia on admission (that is, delta albumin and lymphocyte percentage [ALLY] guidelines) predicts >70% of cases that progress to critical disease [41].

Finally, assays evaluating specific cytokines have further characterized the detrimental inflammatory state in severe COVID-19 and can be used to predict outcomes. Although less commonly measured in clinical settings prior to the pandemic, serum levels of inflammatory interleukins, interferons, and tumor necrosis factor (TNF) have unsurprisingly been associated with active and advanced disease [1, 42, 43]. Namely, interleukin (IL)-6 is one such cytokine that has received much attention as a biomarker and potential targetable effector of COVID-19 severity. IL-6 is a pleotropic factor with numerous immunomodulatory functions. Among its many roles in the pathogenesis and progression of COVID-19 is its involvement in the propagation of COVID-19 cytokine release syndrome, thought to be largely responsible for multiorgan system failure in particularly sick individuals [44]. Indeed, nearly every study with IL-6 data from COVID-19 patients demonstrates levels that correlate with and predict worsening disease outcomes 3, 7, 9, 33, 45]. As with the cellular components likely responsible for its release, IL-6 trajectory is consistently higher over time in individuals that do not survive their disease compared to those who do [2]. To further improve the predictive strength of trending IL-6 by controlling for inter- and intra-patient variability, McElvaney and colleagues developed the Dublin-Boston score, which utilizes the ratio of IL-6 to IL-10, a pro-resolution/anti-inflammatory cytokine, across a 7-day timeline [46]. The authors demonstrated that the Dublin-Boston score outperformed IL-6 measurements alone in predicting clinical deterioration. Although IL-10 is less often measured in the workup and prognostication of COVID-19 and other hyperinflammatory states, this scoring system suggests a potential utility in doing so. The consistent elevation of IL-6 in severe COVID-19 made it an attractive target for intervention, especially given the success of anti-IL-6 agents in other cytokine release syndrome conditions such as in reactions to chimeric antigen receptor (CAR)-T-cell therapy. The efficacy of these medications in COVID-19 remains modest, however, as discussed later in this review.

Ultimately, serum biomarkers obtained from routine clinical laboratories confirm the acute inflammatory state that is expected in a systemic viral infection. Importantly, many of these parameters dependably predict outcomes and offer a pragmatic and affordable tool for clinical decision making. As such, they fill an important void in increasing consistency and equity in the care provided to patients with COVID-19. Where the utility of these measures lessens, however, is in deepening our understanding of the unique pathogenesis of COVID-19 and the mechanisms of its complications. Because the cellular and soluble readouts of these tests look similar in any acute inflammatory state, they do not provide specific mechanistic insights into COVID-19. Instead, more nuanced investigations have been needed. Further, as additional variants of SARS-CoV-2 emerge, the utility and generalizability of biomarker-based prognostication is unclear. A few small studies suggest that while some factors remain predictive irrespective of variant (eg, ALC, neutrophilia), the predictive strength of many inflammatory mediators is lower with more recent variants, especially Omicron [47–49]. As SARS-CoV-2 continues to mutate and adapt, so too will investigators and clinical decision makers need to adjust.

### Leukocyte Population Dynamics Throughout the Acute Phase

As previously described, understanding the relative ratios of lymphocytes to innate immune cells serves as an important initial predictive tool in the progression of COVID-19. Although sufficient to forecast complications, lymphopenia and neutrophilia only provide a superficial view into the immensely complex immune milieu instigated by SARS-CoV-2 infection. In addition to providing important pathogenic information, deeper characterization of leukocyte subsets can also impart further granularity into likely clinical outcomes for patients. To this end, a flood of studies in short succession investigating the immunological nuance in COVID-19 quickly advanced the field's understanding of the significance of lymphopenia in disease progression, which cells contributed to systemic inflammation, and how the balance between hyperactivation and exhaustion dictated patient outcomes.

Central to many of these studies is the question of which T lymphocytes are primarily affected by SARS-CoV-2 infection, and what is the outcome of population restructuring on disease progression. In general, circulating T cells, regardless of subset, are largely depleted from the peripheral circulation in patients with severe COVID-19 [50–52]. The extent and persistence of T-cell reduction correlated with severity of disease and ultimate mortality as patients who survive demonstrate greater total (as well as CD4+ and CD8+) T cells throughout their course [51, 53]. This phenomenon holds true in the response to supportive treatment as well as with steroids and antiviral medications early during the pandemic [51]. Importantly, the T cells that are present in individuals with severe disease demonstrate increased expression of activation and exhaustion markers, providing evidence for one of the leading hypotheses of lymphopenia in COVID-19 [54–56]. This does not appear to be unique to COVID-19, however, as other forms of severe pneumonia are associated with lymphopenia [56]. T-lymphocyte activation phenotypes appear to largely resolve with convalescence as populations from individuals who have recovered from COVID-19 largely resemble healthy donors with regard to activation markers [52]. Recovery from COVID-19 is not without alterations in T-cell populations, however, as convalescence is associated with a striking redistribution of CD4 and CD8 populations from naive to effector memory phenotypes [57]. In addition, a recent analysis of over 4.7 billion T-cell receptor (TCR) sequences across 2130 TCR repertoires from 112 healthy donors and >1600 COVID-19 patients identified TCR signatures associated with disease severity [58]. Notably, a separate study found no evidence for TCR repertoire narrowing over time following repeated antigenic exposures, such as via vaccination or breakthrough infection [59]. Thus, more research is necessary to elucidate the contributions of conventional T cells to SARS-CoV-2 elimination and COVID-19 recovery vs disease exacerbation. Perhaps the ability to elicit a robust and diverse repertoire of virus-specific T cells is a major factor in prevention and/or recovery from severe COVID-19 disease.

Nonconventional T lymphocytes in COVID-19 also exhibit dysregulated functional profiles in COVID-19. As an example, TCR γδ-enriched cells are highly activated as demonstrated by levels of CD38 expression and loss of CD161 in patients with severe disease [56]. And much like their traditional T-cell counterparts, mucosal-associated invariant T (MAIT) cells are largely reduced in patients with COVID-19 and levels appear to be inversely correlated with severity [60–62]. Single-cell RNA sequencing (scRNA-seq) of circulating MAIT cells from COVID-19+ patients segregate from uninfected individuals in unbiased dimensionality reduction, indicating transcriptional differences between the 2 populations [60]. Profiling these cells further reveals a strongly activated phenotype including CD69 and CD56 expression as well as heightened release of IL-6, 8, 10, and 15 [60–62]. Also similar to other T cells, MAIT cells and other unconventional T cells are more likely to exhibit features of exhaustion (eg, PD-1 expression) in individuals with COVID-19 vs healthy donors [62]. Because this activation/exhaustion phenotype occurs in the context of relatively lower frequency, it was again hypothesized that MAIT cells may be experiencing burnout and/or increased migration into peripheral tissues. Interestingly, MAIT cells from COVID-19+ patients have decreased CCR6 expression, suggesting that those cells that remain in circulation may represent those left over from tissue migration [60]. Indeed, bronchoalveolar lavage (BAL) or endotracheal tube aspiration (ETA) from patients with COVID-19 reveals increased MAIT cell presence with a phenotype shifted towards cytotoxicity and pro-inflammatory activation [60–62]. Importantly, a patient's ultimate clinical outcome could be predicted by MAIT cell phenotype: CD69, IFN-gamma, and granzyme B expression all predict poor outcome including mortality [60–62]. Finally, MAIT cells appear to normalize in convalescence, suggesting a dynamic, disease-specific shift in populations between circulation and tissue secondary to disease [61]. Whether the observations of MAIT phenotypes, functions, and association with severity are unique to acute COVID-19 or are present ubiquitously with severe acute infection remains incompletely explored.

Unlike T lymphocytes, B lymphocytes are largely overrepresented as COVID-19 severity increases. In particular, multiple reports have demonstrated that expansion of plasma B-cell populations is predictive of disease severity and negative outcomes [63–65]. This is hypothesized to be due, in part, to preferential expansion of extrafollicular, non-germinal center populations that result in low-selection, low-mutation, SARS-CoV-2-targeting clones derived largely from proliferating naive B cells and not from memory B-cell populations [63, 64, 66–68]. While severe COVID-19 results in a robust, neutralizing antibody response after infection, the ultimate impact of the humoral response is ineffective at clearing viremia and has been associated with worse clinical outcomes. Whether these negative outcomes are secondary to poor viral clearance or from a direct pathogenic effect of the antibodies remains debated; although antibodies secreted from these populations target the receptor binding domain of the SARS-CoV-2 spike protein, they also have been highly associated with autoreactivity and cross-reactions to cellular and extracellular components that can lead to autoimmunity [66–68]. As discussed later, autoantibodies are thought to be a major predictor of COVID-19 complications, and hyperactive extrafollicular plasma cells are one potential source.

In contrast to non-germinal center-derived plasma cells seen in severe COVID-19, studies exploring patients with increased memory B-cell populations or macaque models of seroconversion exhibit markedly improved clinical outcomes, in part through the expansion of germinal centers in the spleen and lymph nodes which, notably, are largely absent in individuals with severe COVID-19 infection [69, 70]. Interestingly, this is also the case in mild COVID-19, whereby memory B-cell responses predominate over extrafollicular B cells activated from naive precursors [71]. A similar phenomenon has been seen when comparing B-cell dynamics in individuals who received an mRNA vaccine against SARS-CoV-2 and those who mounted a humoral response to infection. In contrast to natural infection, mRNA vaccines stimulate formation of robust germinal centers, which are capable of producing antibody responses that are more mature and longer lasting [72]. Further, vaccine-induced humoral responses were much more likely to cross-react with viral variants (while infection-induced antibodies are more transient and are more likely to cross-react with self-antigens). Together, work exploring the heterogeneity of B-cell responses in COVID-19 underscores the importance of memory B-cell-generated antibody responses in effectively controlling viral load, while reactive stimulation of antibody-secreting cells from naive B cells may directly contribute to worsening disease. As an academic prognostic tool, these extrafollicular plasma cells may be used to predict disease outcome or long-term natural immunity, though the utility of identifying these subtypes is clinically infeasible.

Natural killer (NK) cell populations are also substantially altered in COVID-19. As with many other leukocyte populations, NK cells are broadly decreased in individuals experiencing severe disease as demonstrated by classical flow cytometric and scRNA-seq analyses [50–52, 57, 73]. Specific delineation of NK-cell subtypes reveals depletion of antiviral CD56^dim^ cells in ventilator-dependent patients and reduction of CD56^bright^ cells in all infected patients [50, 52, 73]. The activation and exhaustion phenotype theme seen in all T cells and MAIT cells persists with NK cells as well [56, 73]. Ultimately, low levels of total NK cells in circulation are associated with mortality in patients with COVID-19 and patient response to treatment can be, in part, predicted by the rebound of NK cells after treatment initiation [51].

Much like their innate lymphoid cousins, there is substantial alteration to myeloid population frequencies and functions in severe COVID-19 that likely greatly impacts disease course. As discussed above, proportions and absolute counts of neutrophils and eosinophils as measured by CBC analysis are elevated in all patients with COVID-19, with levels rising proportionally with severity of disease [52]. Surprisingly, these cells are not proficiently fighting infection as one may expect, but instead show signs of dysfunction and immunosuppression along with functional indications of impaired oxidative burst and increased expression of CD64 and PD-L1 [74]. This phenotype may be due to immature neutrophil release during emergency hematopoiesis as evidenced by increased levels of pre- and pro-neutrophils on flow cytometry and scRNA-seq analyses [74, 75]. Interestingly, the extent of this hematopoietic response to SARS-CoV-2 infection has led to the discovery of a novel population of *developing neutrophils* in patients with COVID-19 ARDS that appears to exist within a differentiation bridge including plasmablasts [73]. This cell population appears similar to previous studies demonstrating B cell-to-granulocyte/macrophage transitions and is remarkably consistent in samples from patients with ARDS and severe COVID-19 [76].

Monocytes are also substantially altered in COVID-19. Although their absolute numbers are relatively stable, their activation (and thus infection-fighting capabilities) are profoundly diminished as disease severity increases. This is noted with decreased CD16 and HLA-DR expression, along with increased expression of *MAFB, PLBD1*, and *CD163* transcripts [52, 74, 75]. As severe disease progresses with resultant emergency hematopoiesis, proportions of immature monocytes continue to increase. Conversely, patients with mild COVID-19 tend to have early increases in HLA-DR^hi^CD83^hi^ monocytes with strong antiviral interferon gene signature [74]. Among the predictive features of monocyte dynamics, the loss of non-classical monocytes (CD14^lo^CD16^hi^) has been identified as a strong predictor of impending severe COVID-19 [75]. This population transiently decreases prior to severe disease. Such a reduction is not typical of influenza, and an expansion of non-classical monocytes is observed in chronic HIV disease [77].

Multiple groups have used semi-supervised clustering of several immunological parameters to generate *immunotypes* that can more completely predict outcome. The strongest contributing variables to disease-predictive immunotypes include CD4 activation, CD8 cell exhaustion, and non-classical monocyte percentages, among others [53, 54]. Although these methods attempt to reduce the effect of heterogeneity seen even among patients with similar clinical outcomes, there remain countless variables than may impact the existence or power of these immunotypes (eg, timeline of data collection from symptom onset, treatment variability). Further, how widespread vaccination efforts affect the outputs of these methods remains to be seen.

### Multi-omic Characterization of COVID-19 Response and Outcomes

It is clear that as immune cadres rapidly shift throughout the disease course of COVID-19, the clinical utility of pinpointing specific subtypes may be limited. Because disease progression likely occurs secondary to the cumulative influence of molecules released from immunological phenomena *in toto*, identifying circulating factors downstream of the immune system may prove beneficial in predicting progression and targeting therapies. Just as multiplexed flow cytometry and scRNA-seq provided advanced alternatives to the CBC in identifying effector cells, investigators have utilized multi-omic screens to identify novel markers of COVID-19 outside of those found in traditional clinical chemistry panels. While some groups utilized a completely unbiased approach with mass spectrometry (MS)-based proteomic and metabolic methods, others utilized what was known about the pathogenesis of COVID-19 to tailor large soluble biomarker screens. In one such study, a panel of 64 proteins related to inflammation, coagulation, and tissue damage was measured longitudinally in patients either positive or negative for COVID-19, along with a spectrum of disease severities within those groups [78]. As predicted, numerous individual bio-markers were associated with poor prognosis including inflammatory cytokines (eg, IL-6, IL-8, TNF), pro-coagulant factors (eg, tissue factor, urokinase plasminogen activator receptor [uPAR]), and markers of endothelial activation (eg, VCAM-1, RAGE). Importantly, the trajectory over time of these (and other) markers was also predictive of outcomes just as IL-6 is, as previously discussed. In addition to being associated with ICU need, these factors were also predictive of specific organ dysfunction such as venous thrombosis/pulmonary embolism and renal replacement therapy need. To identify specifically enriched pathways that predict negative outcome from this panel, the investigators performed pathway analysis on all of the biomarkers and found that outcomes of ICU patients were associated with chemotaxis, the complement system, innate host response systems, among others [78]. Importantly, the specific proteins selected for the panel were those belonging to these pathways, so the exact utility of pathway analysis in this instance can be debated. Regardless, multiple high-throughput studies have recapitulated the importance of these pathways: using untargeted MS on plasma from COVID-19+ patients across WHO clinical severity groups, Messner et al identified 27 protein biomarkers whose levels change in correlation with disease severity, using multiple different patient datasets [79]. These proteins, many of which had not yet been associated with COVID-19 pathogenesis, belonged to pathways such as the complement system, coagulation, and interleukin signaling. Perhaps even more important to the progress of COVID-19 biomarker identification, this study and others have described low-cost, high-throughput, highly reliable screening and validation protocols across samples from different institutions, stored under different conditions [79, 80].

Just as temporal information of CBC or plasma chemistries increases their utility as predictive tools, measuring the plasma proteome over a patient's disease course allows investigators to build models to predict outcomes more accurately. By combining a large collection of clinical measurements, standard clinical laboratory tests, clinical risk scores, and untargeted MS-proteomics for patients across multiple timepoints, Demichev and colleagues were able to generate a remarkably integrated dataset for each patient that could be associated with their clinical course [81]. After establishing such a dataset for 139 patients across 687 sampling points, they were able to utilize machine learning to create a predictive model that is able to predict disease outcome; the need for renal replacement therapy, extracorporeal membrane oxygenation, and invasive ventilation; they also identified age-related patterns of disease. As with other reports, the vast majority of proteins identified using proteomics that contributed to disease outcome within the combined model were those related to inflammation and complement activation.

The utilization of alternate energy sources and regulation of metabolic pathways (so-called immunometabolism) is also critical for an efficient immune response to SARS-CoV-2. Indeed, using screening proteomics and metabolomics followed by targeted validation assays, Shen et al identified 93 differential proteins and 204 differential metabolites that correlated with disease severity [82]. Pathway analysis of these factors identified a strong enrichment for complement activation, platelet degranulation, and acute phase proteins. Interestingly, metabolomics identified numerous lipids dysregulated in COVID-19+ patients, while the study by Demichev et al identified differential levels of apolipoproteins [81]. Together, these studies underscore the massive metabolic impact the immune response to SARS-CoV-2 infection requires and may explain the importance of pre-infection metabolic status organs such as the liver.

### Autoantibodies in COVID-19: Predilection or Reaction to Disease?

Among the more surprising discoveries coming from research into the first wave of COVID-19 was the finding that autoantibodies were often found in patient sera during the acute infection, and that certain autoantibodies were highly correlated with COVID-19 morbidity and mortality [83–86]. While the significance of this finding is debated and likely depends on specific antibody species formed, the heterogeneity of responses with regard to autoantibody production makes it potentially useful in further stratifying/characterizing specific patient groups. As with protein biomarker discovery, investigators have used targeted and untargeted approaches to quantify and characterize autoantibodies in patients with COVID-19+. In a high-throughput screen of 172 patients across clinical severities (along with asymptomatic and COVID negative individuals), one group identified numerous putative autoantibodies using rapid extracellular antigen profiling (REAP) [87]. Remarkably, the authors found that patients with COVID-19 had a higher composite REAP score than patients with systemic lupus erythematous, a condition defined by widespread autoantibody production. As noted previously, the targets for autoantibodies in COVID-19 runs the gamut of antigens, though those targeting immune functions such as interferons and other cytokines and chemokines appear particularly enriched [85, 87]. Of note, the prevalence of antibodies targeting type I interferons ranges from 5% to 10% of patients with COVID pneumonia depending on the study, illustrating how adequate viral clearance may remain elusive. Importantly, auto-antibodies against cytokines, chemokines, and their receptors are often functionally active, capable of inhibiting signaling pathways ex vivo and can worsen disease in a murine model of COVID-19 [87]. Interestingly, investigation into the temporal dynamics of identified autoanti-bodies revealed a proportion of antibodies that were likely present pre-infection (including those against IFNα2 and IL1β), illustrating the complex nature of whether pre-existing autoantibodies increase susceptibility to infection and progression of COVID-19 or whether they result from the infection itself. The presence of antibodies targeting type I interferons, for instance, greatly amplifies COVID pneumonia, increasing patient risk for severe illness and targeting these antibodies with plasma exchange can improve recovery [84, 86]. Further, multiple studies have demonstrated that these antibodies often pre-date infection with SARS-CoV-2 and increase risk of severe COVID-19 [84, 88, 89]. In fact, a recent study demonstrated that 5% of uninfected individuals over 70 years old have autoantibodies targeting type I interferons, yet they are associated with 20% of COVID-related deaths in aged patients [83]. Complicating this hypothesis, however, are the observations that early humoral responses to SARS-CoV-2 infection generate cross-reactive antibodies that simultaneously target the viral pathogen and autoantigens such as those seen in systemic lupus erythematosus (SLE) [66, 67]. As such, some autoantibody species are likely reactionary to infection and do not represent a predisposing risk factor. While the presence and cross-reactivity of these antibodies after infection have been well characterized, their causal impact on COVID-19 complication occurrence have not been explicitly demonstrated.

As mentioned above, numerous other potentially pathologic autoantibodies have been identified in patients with COVID-19 including those against angiotensin II, neutrophil extracellular traps, and numerous factors seen in systemic autoimmune and connective tissue diseases (eg, SLE, systemic sclerosis, myositis) [90–94]. In many cases, the temporal and causal relationship between these antibodies and disease remains logistically difficult to ascertain.

Another auto-immune process that has garnered attention are the pathogenic antibodies seen in antiphospholipid antibody (aPL) syndrome (APS). These families of antibodies, including anticardiolipin, anti-β_2_ glycoprotein, and anti-phosphatidylserine/prothrombin, have been identified in nearly half of all patients with COVID-19, depending on the study [95–97]. Given the propensity of these antibodies to induce catastrophic thrombosis in the non-COVID-19 setting, similar pathways could contribute to thrombotic complications during or after COVID-19. However, whether aPLs are a contributor or bystander remains uncertain [95, 96, 98, 99].

### Tissue-Based Biomarkers: Beyond the Serum

While serum/plasma-based studies have been the most accessible and pragmatic options for rapid biomarker identification, efforts to analyze tissue-level effects have also been successful. Consistent with findings in the blood, BAL fluid from patients with eventual decompensation demonstrated a neutrophil predominance and diminished interferon response [100]. Further, BAL fluid/blood ratios of inflammatory cytokines are decreased in patients with worse outcomes. A post-acute lymphocyte predominance in BAL fluid may contribute to long-lasting post-COVID symptoms (discussed below) [101]. As with serum studies, investigators have also used unbiased proteomics approaches to identify differential proteins within BAL fluid that may eventually be used for targeting therapies [102]. Interestingly, biomarker identification within the lungs is not limited to cellular and protein levels: exhaled nitric oxide (NO) concentrations have been identified as surrogates for distal lung inflammation and are predictive of disease severity, which is concordant with recent efforts to use inhaled NO as an anti-inflammatory agent in COVID-19 [103, 104].

Biomarker measurements from the urine and stool can also identify relevant immunologic processes [105–107]. Further, in patients needing a lumbar puncture to evaluate acute encephalopathy, biomarkers identified in cerebrospinal fluid such as neurofilament light chain (NfL) and inflammatory cytokines may be of some utility [108, 109].

Early in the pandemic, lung imaging observed abundant ground-glass opacity in those with severe disease. In the years since, multiple investigators have used large, multicentered cohorts of chest CT images to develop models that can correctly diagnose COVID pneumonia and predict its progress and outcome [110–113]. This work eventually led to the creation of a radiomic nomo-gram that can be applied to heterogenous institutions to predict the need for invasive ventilation [114]. Further underscoring the systemic effects of COVID-19, recently published work created a pipeline that utilizes chest CTs to estimate hepatic steatosis which could eventually predict COVID-19 severity across multiple organ systems (eg, extracorporeal membranous oxygenation use, invasive ventilation, mortality) [115]. Altogether, radiomics not only has the potential to positively impact individual patient care, but also can assist at the institution level in resource allocation during particularly inundating waves.

The power of biomarkers collected at the time of presentation or throughout the disease course exists in their ability to predict the presence of COVID-19 and how it may progress. As detailed above, hundreds of studies have established convincing evidence for targeted and high-throughput assays in identifying such factors in tissues or images with the hopes of predicting outcomes and affecting patient care. Anecdotally and as demonstrated by the low rates of death among those triaged to home with COVID-19, frontline providers have demonstrated an incredible ability to predict these very same features in their patients. Prospective studies also suggest that clinician gestalt is often non-inferior to objective risk scores created using clinical parameters and biomarker data, underscoring the importance of provider-patient interaction during initial presentation of disease [116, 117].

### Anticipatory Immunologic Features: Protecting At-Risk Populations

Biomarkers of the acute response to infection require, by their definition, a patient to be infected with a pathogen and mount a physiologic and/or immunologic response to that infection. Because of this, clinicians and guideline writers are forced to play defense as they respond to an actively evolving immunological process. If, instead, pre-infection biomarkers were identified that could guide preventative efforts against infection in high-risk individuals, the healthcare system could go on the offensive in the fight against spread and mortality. Fortunately, such risk factors and serological markers have begun to be discovered and were essential in initial policies surrounding isolation, masking, and vaccination roll out. One biomarker that increases risk for severe COVID-19 already discussed is the existence of autoantibodies against the type I IFN system, although specific interventions surrounding the presence of these with regards to prevention have not yet been explored. Within this section, we discuss disease states and circulating factors that may predict risk of infection and disease progression, highlighting the future of how anticipatory biomarkers can be leveraged to protect and screen vulnerable populations.

### Clinical Comorbidities

Early epidemiological studies during the first wave of COVID-19 quickly identified conditions and risk factors that increased susceptibility to infection and/or worsening of disease. Although not biomarkers, per se, understanding these associations was critical in protecting at-risk individuals through vaccination efforts. Among the clinical states with the highest risk for severe COVID-19, chronological age remains one of the strongest predictors even when analyses control for other age-associated comorbidities [118, 119]. The mechanisms by which aging affects infection risk and disease outcome are likely numerous with a cumulative effect reflecting multiple measurable and unmeasurable processes. Among these include profound dysfunction of the immune system secondary to senescence and progenitor cell burnout, as well as end-organ physiology dysfunction (eg, diminished respiratory function at baseline, increased susceptibility to cardiovascular decline) [120–122]. Other biological/pathological conditions associated with heighted risk of COVID-19 decompensation include chronic lung disease (eg, asthma, COPD, ILD), cancer (of all types), chronic kidney disease, chronic liver disease, diabetes, ischemic and non-ischemic heart disease, obesity, immunodeficiency (eg, HIV, post-transplant immunosuppression), mental health conditions, and neurologic conditions [123]. Importantly, the risk ratio of death per decade of life is far higher than for any individual medical comorbidity and the more comorbidities an individual has, the higher their risk [124, 125]. Interestingly, for each of these conditions, there is not a consensus regarding mechanisms by which the disease increases risk for COVID-19 severity.

Among many other lessons learned, the COVID-19 pandemic has underscored systemic failures by the healthcare system and society to achieve health equity. Individuals of marginalized racial, ethnic, and socioeconomic groups have been consistently observed to have higher infection rates and more severe outcomes [126]. Thus, studies that address how social and biologic determinants of health may intersect and lead to health disparities are increasingly relevant to the health of individuals and societies as a whole.

### Genetic Variants of COVID-19 Susceptibility

Given the complexity of mechanisms at play in COVID-19, it should not be surprising that many genetic traits can influence risk. Indeed, monogenic and polygenic loci have been identified which increase susceptibility to infection and severity. In an impressive display of international collaboration within the scientific community, multiple studies through the COVID-19 Human Genetics Initiative along with other more localized consortia uncovered associations between several individual variants and critical disease in COVID-19 [127–129]. Among the identified loci, many were associated with immunologic processes including chemokine receptors and type I interferon signaling. Polygenic risk scores used to predict COVID-19 severity also implicate polymorphisms in genes related to immune function, underscoring the dysregulation of immunologic processes in severe COVID-19 [130, 131]. Taking the totality of these variants and applying them to a Phenome-Wide Association Study (PheWAS) within the Million Veterans Program database, Verma et al identified a striking association between COVID-19 outcomes, genetic variance, and comorbidities known to increase risk for COVID-19 severity (detailed above including chronic lung disease, autoimmune conditions, immunosuppression) [132]. In general, these studies further illustrate the heterogeneity in immunologic function which exists prior to infectious exposures, and many are consistent with the wide range of clinical outcomes and variant immunophenotypic and proteomic observations made after infection. The biological processes identified in these studies can also provide essential target validation to support the design of therapeutics (see below).

### Chronic Viral Carriers: Priming Immunohematologic Reactivity

Importantly, SARS-CoV-2 infection and its subsequent impact on the antiviral functions of the immune system often do not exist in a vacuum. As such, another anticipatory factor that can impact COVID-19 outcomes is an individual's serologic status to other chronic viral infections. Among these, cytomegalovirus (CMV) appears to pose a unique and significant risk towards COVID-19 symptomatology and progression. One retrospective study investigating CMV seropositivity in German patients found that CMV positivity was associated with higher rates of severe COVID-19, especially in populations without co-existing clinical conditions [133]. A study analyzing IgG titers against CMV in pre-infection serum samples suggest prior CMV seropositivity was associated with an odds ratio of 1.7 for SARS-CoV-2 infection after adjusting for age, sex, and race [134]. Further, CMV seropositivity was also associated with increased likelihood of hospitalization after infection without evidence of latent CMV reactivation. Mechanistically, the authors demonstrate that CMV seropositivity is associated with expanded and activated effector memory T-cell populations, which, along with heightened innate immune cell activation seen in CMV infection, could contribute to the hyperinflammatory state seen in severe COVID-19. While CMV reactivation was not demonstrated in this study, other investigators have shown a modest awakening from latency in COVID-19 patients that may contribute to hyperinflammation as well [135]. In addition, latent CMV infection may predispose individuals to reduced neutralizing and anti-spike antibody titers following primary-series SARS-CoV-2 mRNA vaccination, even after adjustment for age, sex, and race [136].

Epstein-Barr virus (EBV) has also been identified as a potential latent infection that influences an individual's response to COVID-19. As with CMV, EBV reactivation has been identified in small subsets of critically ill patients, signifying an additional potential cause of the widespread inflammatory response [135]. Other small retrospective studies and case reports have described increased inflammatory markers in EBV/SARS-CoV-2 co-infection and also reactivation of EBV after SARS-CoV-2 infection that increases risk of death [137–139]. Reactivated EBV infection has also been linked to post-acute sequelae of COVID-19 (PASC). Additional viral species may also shape acute and convalescent processes after SARS-CoV-2 [140–145].

Several studies suggest that previous infection with other, seasonal, human coronaviruses (hCoV) appears to result in cross-reactive T cells and may provide some protection against SARS-CoV-2 [146]. These T cells are readily detected in samples acquired prior to the pandemic and their presence has been associated with improved vaccine T cell and antibody reactivity to low-dose mRNA vaccination [147]. Conversely, other studies describe long-lasting hCoV antibodies that do not cross protect against SARS-CoV-2 but, instead, increase susceptibility to infection, reduce efficacy of mounting a humoral response to SARS-CoV-2, and ultimately worsen COVID-19 severity [148, 149]. These phenomena, paired with the observation that hCoV often interact and coinfect with other respiratory viruses, may explain the 2022-2023 emergence of frequent SARS-CoV-2 and respiratory syncytial virus co-infections [150]. In addition to providing information regarding a particular individual's risk for severe COVID-19, understanding serology against hCoV and other respiratory viruses prior to and at the time of SARS-CoV-2 infection may continue to aid in understanding determinants of an efficient protective adaptive immune response to SARS-CoV-2.

### Lessons From Interventional Trials

While risk stratification and understanding the pathogenesis of COVID-19 are often the primary objectives of biomarker research, multiple clinical trials have sought to determine the extent to which immune pathways are causal to adverse clinical events, albeit with mixed success. Given the near ubiquitous identification of IL-6 as a powerful predictor of disease course in initial studies, along with its involvement in other disorders of cytokine storms, IL-6 and its signaling pathways became logical targets in severe COVID-19. As trials using monoclonal antibodies targeting IL-6 receptor (IL-6R) (eg, tocilizumab, sarilumab) or IL-6 itself (eg, siltuximab) began to conclude, it was clear that the utility and efficacy of IL-6 blockade was dependent on numerous factors such as time of administration, severity of disease, and concomitant immunomodulatory agents [151–153]. A meta-analysis of several studies targeting IL-6 suggests a modest favorable overall effect on mortality and other outcomes [154]. Caution may be warranted as there is evidence that inhibiting IL-6 activity can negatively impact neutralizing antibodies against SARS-CoV-2 [155].

Another potent cytokine induced during the hyper-inflammatory state of COVID-19 is interleukin-1 beta (IL-1β) [156]. However, as with IL-6, results from trials of IL-1β blockade (eg, anakinra, canakinumab) in COVID-19 were also conflicting, potentially due to the timing of initiation and extent of inflammatory disease [157]. When started early in the course, but when inflammation was already elevated (as evidenced by elevated uPAR levels), anakinra improved clinical course and mortality [158]. Similar effects were noted in a meta-analysis of anakinra trials using CRP as a surrogate for inflammation [159]. Conversely, the benefit of initiating IL-1β blockade may be attenuated when initiated later in the clinical course, illustrating the complex temporal dynamics of the immunologic response as detailed previously.

Colchicine also failed to convincingly demonstrate clinical benefit in community-based or hospitalized settings, once again demonstrating the challenge of optimizing the inflammatory milieu in this disease [160–162]. These trials illustrate the fine balance between the protection and risk conferred by pro-inflammatory pathways elicited during the acute response to SARS-CoV-2.

The therapeutic potential of boosting the immune activation by administering exogenous type 1 interferons is also supported by early biomarker studies. Early efforts utilizing interferon-alpha (IFNα) or interferon-beta-1 (IFNβ1) demonstrated no significant reduction in disease severity when administered alone or in combination with antiviral medications (eg, remdesivir) [163–165]. When administered early and in non-critical patients, however, there appear to be modest benefits in mortality [166, 167]. Further, as part of a *triple-therapy* regimen consisting of lopinavir-ritonavir, ribavirin, and IFNβ1, the addition of interferon therapy reduced symptom duration and hospitalization in patients with mild and moderate COVID-19 [168]. Ultimately, as with other immunomodulating interventions, early administration may be key to the efficacy of interferon therapy [169].

Finally, inhibitors of cytokine signaling pathways such as the JAK/STAT inhibitors (eg, baricitinib, ruxolitinib, and tofacitinib) and non-specific tyrosine kinase inhibitors (eg, imatinib) have also shown promise in modulating the immune response in COVID-19. [170] Among these, barcitinib has had 2 large, randomized control trials demonstrating its efficacy in reducing hospitalization, mortality, and other serious sequelae [171, 172]. These effects were especially prominent in patients with severe disease. Interestingly, one meta-analysis comparing immunomodulatory clinical trials revealed that JAK/STAT inhibitors were highly efficacious at achieving protection from mortality due to COVID-19 along with posing less risk on superimposed infection [173]. Altogether, therapies targeting the immune response to SARS-CoV-2 have achieved mixed success, but it is clear that rapid patient identification, risk stratification, and therapy delivery may be paramount to re-programming the immune system in the setting of acute COVID-19.

### Future Directions

Although vaccination efforts have been remarkably successful thus far in reducing severe COVID-19 cases, ongoing biomarker research is needed to predict acute and chronic manifestations of disease and to identify novel targets for treatment of breakthrough severe cases. This is especially salient as new variants arise and the longevity of inoculated immunity remains to be seen. Long-term outcomes and complications of COVID-19 are only now beginning to be understood as many patients infected during the initial wave are presenting with symptoms of PASC. While identified mediators of PASC mirror many of those at play in the acute phase of COVID-19 (eg, inflammatory cytokines, autoantibodies, chronic EBV), predicting outcomes of this unique post-viral syndrome remains complex [174–176].

The wide divergence in acute outcomes (6% fatal, ~25% asymptomatic in the first wave) of COVID-19 and the unpredictable nature of symptomatic recovery highlight arguably the most important scientific challenge for the post-pandemic era. As a new global exposure, SARS-COV-2 was able to dramatically demonstrate at the clinical level that which immunologic biomarker research has been suggesting for decades — an incredible variation in immune function seems to exist, even among seemingly healthy adults. The implications of this are that greater emphasis may be needed in understanding *baseline* variation in populations, and that immunotherapeutic studies may need to be planned and properly powered with *the expectation* that heterogeneity in treatment response may be encountered. As such, concerted efforts are needed to continue large-scale, high-throughput biomarker discovery so that population-level predictions can be made, but also identify relevant endotypes. Further, understanding how biomarkers can be used for populations with specific comorbidities will allow more personalized care guidelines and protocols to be developed should an individual become infected. Finally, continuing to explore anticipatory biomarkers using inexpensive, routine clinical tests or exam parameters that can be easily mobilized to diverse communities will represent small steps in reducing the impact of socioeconomic determinants on COVID-19 outcomes.
